# A Case of Pregnancy Complicated with ATIII Deficiency in a Patient Who Developed Severe Venous Thromboembolism in Her Fourth Pregnancy and Had a Favourable Outcome in Her Subsequent Pregnancy with Careful Management of Anticoagulation Therapy including Edoxaban

**DOI:** 10.1155/2019/2436828

**Published:** 2019-01-02

**Authors:** Mie Sakai, Jumpei Ogura, Koji Yamanoi, Takahiro Hirayama, Tsutomu Ohara, Haruka Suzuki, Yoshihide Inayama, Koji Yasumoto, Koh Suginami

**Affiliations:** Department of Obstetrics and Gynecology, Toyooka Public Hospital, Japan

## Abstract

Congenital ATIII deficiency is one of the congenital thrombophilia diseases that can cause severe venous thromboembolism (VTE) in pregnant patients. A 30-year-old female, 4 gravida and 2 para, came to the emergency department with a complaint of oedema and pain in the left lower leg at 11 weeks of gestation. An inferior vena cava thrombus and pulmonary embolism were found. Because VTE was very severe, artificial abortion was performed, and VTE disappeared rapidly. She maintained oral administration of edoxaban (NOAC) and got pregnant naturally fifty-five weeks later after the abortion. Anticoagulation therapy was changed from NOAC to ATIII formulation and unfractionated heparin at 5 weeks of gestation. The course of pregnancy was good, and a healthy female newborn of 2310 g was delivered vaginally at 37 weeks 6 days of gestation. In puerperium, anticoagulation therapy was changed to warfarin. Currently one and one-half years had passed after delivery and no major adverse events or thrombosis has occurred. This case indicates that severe VTE can develop even in multipara pregnancy and that those who take NOAC may be able to continue pregnancy when they get pregnant.

## 1. Introduction

Venous thromboembolism (VTE) is quite an important disease because it sometimes directly relates to severe events. It is well known that pregnancy itself is one of the risk factors of VTE and is one of the major reasons of maternal death during pregnancy and postpartum [1–3]. If patients have hereditary thrombophilia disease, risk of VTE during pregnancy rises markedly [4–8].

Congenital ATIII deficiency is one of the autosomal dominant congenital thrombophilia diseases. Mutation of more than 250 ATIII-related genes suppresses transcription and decreases the antigen amount or activity, thereby decreasing the antithrombotic action and frequently causing thrombosis [9, 10]. The mutation was first reported by Warren et al. [11] in 1935, and the incidence rate is said to be approximately 1/2500 [10]. Thrombosis often presents as an initial symptom in young ATIII-deficient patients between the ages of 10 and 35, and it is well known that its frequency increases markedly during pregnancy [12–15]. However, not all ATIII-deficient patients have a family history. Furthermore, because not all ATIII-deficient patients develop VTE during pregnancy, it can happen that VTE does not develop at their first or second pregnancy.

Here, we present the case of a patient who developed severe VTE and was diagnosed as congenital ATIII deficiency in her fourth pregnancy. Although she was forced to undergo an artificial abortion because of the seriousness of VTE, she got pregnant naturally with administration of edoxaban (NOAC). In her fifth pregnancy, we cooperated closely with cardiovascular doctors, performing prophylactic anticoagulant therapy, and she delivered a healthy baby.

## 2. A Case Report

### 2.1. Development of Severe VTE and Treatment and Diagnosis of Congenital ATIII Deficiency

The patient was a 30-year-old woman (4 gravida, 2 para, and 1 artificial abortion) who got pregnant naturally and came to our department. She had no family history of VTE. She had no major illness in the past. Her menstrual cycle was approximately 30 days regularly. At 24 years old, she delivered a male baby of 2900 g vaginally at 40 weeks of gestation. In addition, at 27 years old, she delivered a female baby of 2345 g vaginally at 38 weeks of gestation. She is a nonsmoker and nonalcoholic drinker.

**Figure 1:**
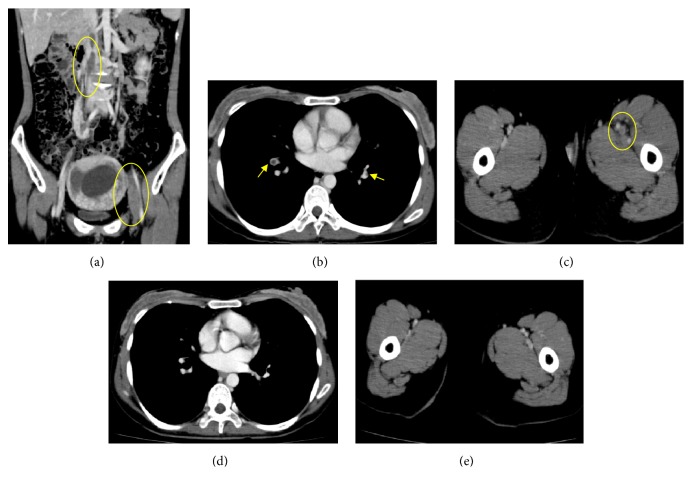
Development of severe VTE, and its disappearance after treatment at patient's fourth pregnancy. (a~c) Contrast CT image taken at the admission day. (a) Coronal plane, large thrombosis exists in inferior vena cava and femoral vein. (b) Transverse plane at the level of trunk of pulmonary artery. (c) Transverse plane at the level of femoral vein. Yellow circle and arrow indicate the location of venous thrombosis. (d, e) Contrast CT image taken at the start of oral administration of edoxaban. (d) Transverse plane at the level of trunk of pulmonary artery. (e) Transverse plane at the level of femoral vein.

At 11 weeks of gestation, she presented to the emergency department of our hospital with a complaint of edema and pain in the left lower leg. Because VTE was strongly suspected, a contrast-enhanced computed tomography (CT) examination was performed with the consent of the patient. Then, a bilateral peripheral pulmonary embolism and venous thromboembolism from the left femoral vein to the inferior vena cava (renal vein level) were detected (Figures 1(a), 1(b), and 1(c)).

The IVC filter was placed emergently, and anticoagulation therapy with continuous heparin infusion started immediately. A blood examination taken at the day of admission revealed a decrease in protein S activity (33%) and ATIII (54%) (Table 1(a)). Those findings strongly suggested the existence of some congenital thrombophilia disease, such as protein S deficiency and ATIII deficiency. Because VTE was very severe, the cardiologists recommended a termination. With consent of the patient and her family, followed by enough discussion with us and the cardiologists, an artificial abortion was performed at 15 weeks of gestation. After surgery, VTE improved dramatically and finally disappeared (Figures 1(d) and 1(e)). The IVC filter was removed, and the anticoagulation therapy was changed to oral administration of NOAC. Then, she was discharged from the hospital.

After discharge, we repeated blood examination and found that the ATIII value maintained at a low level, 33.9% (Table 1(b)). Further detailed examination was performed in another hospital, and congenital ATIII deficiency was diagnosed.

### 2.2. Management of Antithrombosis Therapy during Subsequent Pregnancy

The patient strongly hoped for pregnancy. Followed by detailed explanation to her and her family, we decided to maintain NOAC treatment and allowed her to become pregnant.

**Figure 2:**
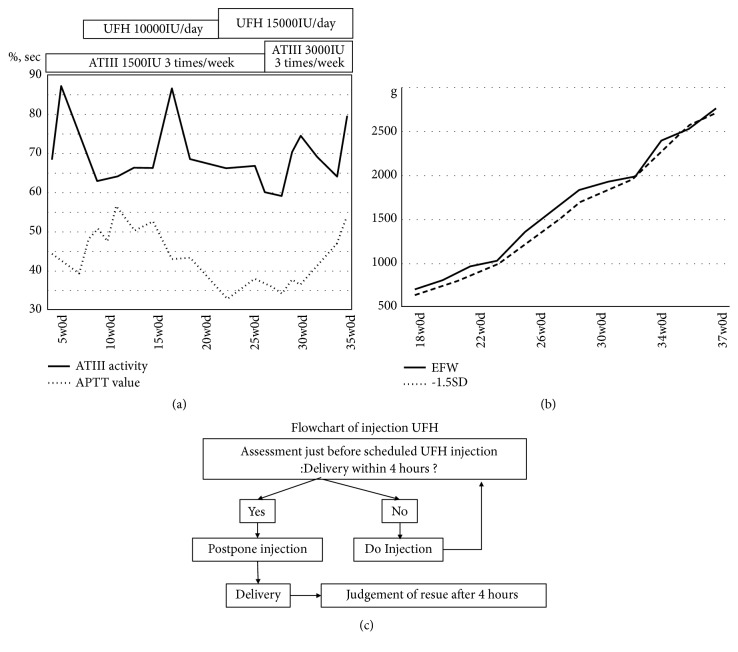
Clinical course in patient's fifth pregnancy, and flow chart in delivery. (a) Treatment process and change of ATIII activity and APTT value during pregnancy. Y-axis; % (ATIII) and seconds (sec, APTT). (b) Change of the estimated fetal weight (EFW) of fetus. Dot line indicates the line of -1.5SD. (c) Flowchart that we considered at the delivery.

Fifty-five weeks after the abortion, she came to our department with a complaint of 4 weeks of amenorrhea, and a gestational sac was detected by ultrasound examination. At that time, there were no symptoms of thrombosis clinically. We immediately changed anticoagulation treatment from NOAC to a regular administration of ATIII formulation. To keep ATIII value above 70%, we appropriately changed the dose and frequency (Figure 2(a)).

Although no thrombosis was detected by ultrasound examination, we thought it was better to conduct administration of unfractionated heparin (UFH) at the same time. To maintain an APTT value of approximately 50 to 60 seconds, we appropriately changed the dose and frequency (Figure 2(a)). During pregnancy, no symptoms of thrombosis were observed.

The development of the baby was observed carefully by ultrasonic examination. Foetal estimated body weight was from -1.5 to -1.0 SD during pregnancy (Figure 2(b)). No obvious anomaly was detected, and amniotic fluid volume was within normal range. There was no obvious abnormality in the blood flow of the umbilical artery and middle cerebral artery. Foetal heart beat monitoring was observed once a week from 36 weeks of pregnancy, and there were reassuring patterns.

### 2.3. Management of Delivery

As for delivery, we decided to have a planned vaginal delivery. We discussed with the cardiovascular doctors and paediatricians and planned a delivery as below (also, a flowchart is shown in Figure 2(c)).

At term, UFH was administered at fixed times, 5:00, 13:00, and 21:00. If delivery was expected within 4 hours, UFH would be postponed. The ATIII formulation would be administered on schedule. After delivery, we would resume UFH 4 hours later and return to the scheduled subcutaneous injection.

At the time of admission (37+5 weeks of gestation), internal examination revealed that the cervix opened to 2.5 cm in dilatation. A balloon catheter (120 ml of distilled water) was inserted at noon. An irregular uterine contraction started approximately 14:00. At 21:00, the cervix dilated to 8 cm. We expected that delivery would be within 4 hours, and APTT value at that time was prolonged to 59 seconds. The scheduled administration of UFH was postponed.

The delivery gradually progressed. At 0:58, a baby of 2310 g was delivered vaginally with Apgar score 9/9 and an umbilical artery pH of 7.352. The total blood loss during delivery was 420 ml. At 5:00, we resumed administration of UFH because 4 hours after delivery had passed, and no symptoms of bleeding tendency were observed. The amount of vaginal bleeding did not increase after resuming UFH.

We gradually switched ATIII and UFH to warfarin from the second day of postpartum.

Warfarin was started from 3.0 mg / day and was gradually increased until the PT-INR reached the range of 2.0 to 3.0. At the same time UFH was gradually decreased from 15000 units/day. We changed the dose of reagents very carefully so as not to cause any complications. Since PT-INR was kept within 2.0 to 3.0 by only oral administration of warfarin from the 11th day of postpartum, she was discharged from the hospital on the 14th day of postpartum. Currently about one and one-half years had passed since delivery, and she has maintained warfarin treatment without development of VTE.

**Figure 3:**
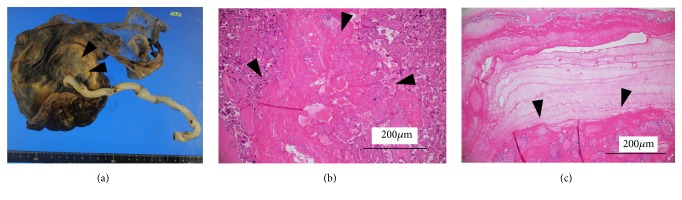
Pathological findings of placenta. Macroscopic view and HE staining are shown. Scale is shown in each figure. (a) Macroscopic view; circumferential-like infarction was observed in a portion approximately 4 cm in radius around the umbilical cord. (b) x40 magnification. Necrosis of villi was observed around that site. Black arrows indicate the necrosis of villi. (c) x40 magnification. Necrosis of the amniotic membrane and chorion, and fibrin precipitation was observed. Black arrows indicate the necrosis, and fibrin precipitation.

Because the baby's weight was less than that of the 10th percentile, we examined the placenta pathologically. The placenta weight was 486 g, which is relatively smaller than that of average. Macroscopically, a circumferential-like infarction was observed in a portion approximately 4 cm in radius around the umbilical cord (Figure 3(a)). Necrosis of the amniotic membrane and chorion was observed microscopically at that site. In addition, necrosis of villi and fibrin precipitation was observed around that site (Figures 3(b) and 3(c)). These findings are probably due to the influence of congenital ATIII deficiency.

## 3. Discussion

This time, we experienced a case of congenital ATIII deficiency, which was first diagnosed when development of VTE was observed at her fourth pregnancy.

Congenital ATIII deficiency is one of the hereditary thrombophilia diseases, and it is known that the risk of VTE is very high especially during pregnancy. Charles et al. report that the VTE rate of pregnant women who do not have a history of VTE is 3-7%, while that of pregnant women with congenital ATIII deficiency is 40%, which is 6-13 times higher than that in normal pregnant women [10]. However, in this case, she had no family history or medical history of VTE and developed VTE for the first time during her fourth pregnancy. Although the risk of VTE in congenital ATIII deficiency patients during pregnancy is extremely high, VTE does not develop in all patients. In addition, such as this case, patients do not always have family history or medical history of VTE. When severe VTE is detected during pregnancy, even if she has no family or medical history and is multipara pregnant, the presence of some hereditary thrombosis should be taken into consideration.

There is a domestic guideline for the prevention and treatment of VTE during pregnancy in Japan. For VTE high-risk pregnancies, preventive anticoagulation with UFH is recommended, and in the case of ATIII deficiency, it is recommended to add the use of ATIII formulations. In some other countries, we can see more reports using low molecular weight heparin (LMWH) for pregnant women compared to those using UFH. ACCP guidelines mostly recommend LMWH as a first choice for anticoagulation therapy for pregnant women [16]. Some retrospective analyses and systematic reviews show that the risk of bleeding during pregnancy due to LMWH administration is low compared to that due to UFH [17, 18]. In addition, because UFH can maintain the anticoagulant effect for a long time, management of UFH during delivery may be slightly more complicated compared to LMVH. However, in Japan, we are currently not allowed to use LMWH with public insurance. We hope that we can use LMVH for pregnant women in the near future.

At the delivery during antithrombotic therapy, we should pay attention to the adverse effects of both bleeding tendency caused by antithrombotic therapy itself and development of thrombosis caused by interruption of antithrombotic therapy. In this case, we devised a flowchart (Figure 2(c)) to prevent administration of UFH within at least 4 hours before delivery, since UFH was administered every 8 hours. We also had a close meeting with ward staff, midwives, paediatricians, and anaesthesiologists in advance.

Foetal growth restriction (FGR) is frequently observed in the patients with congenital AT III deficiency [19]. In this case, the estimated foetal body weight of her baby was relatively small compared to average, about -1.5 to -1.0 SD, but the amount of amniotic fluid was normal. The baby's birthweight was 2310 g, which was below the 10th percentile and was defined as small for gestational age (SGA). In the pathological examination of the placenta, the infarction existed in a relatively wide range, suggesting a thrombotic tendency due to ATIII deficiency, which might be the reason of SGA.

In this case, the patient got pregnant naturally during the oral administration of NOAC. NOAC is currently preferred to UFH and/or LMWH for nonpregnant patients from the viewpoint of administration route and management. Nonetheless, the influence of NOAC on pregnancy has not been established today. Hence, we should use UFH or LMWH during pregnancy, not NOAC. Burnet et al. have summarized cases taking NOAC in early pregnancy [20]. In their report, among 10 pregnant women who were exposed to NOAC during the first 6 weeks of gestation, 4 were full-term births, 2 were preterm births, 1 was a first-trimester spontaneous abortion, and 3 were elective pregnancy terminations. NOAC animal studies demonstrated no fetal harm. In addition, they say that NOAC is not contraindication to those who are pregnant. In this case, the patient was exposed to NOAC during the first 5 weeks of gestation, and she had a full-term birth without any anomaly in her baby. Evaluating the influence of NOAC on pregnancy is highly recommended in the near future. We hope we will be able to use NOAC even during pregnancy.

In conclusion, we experienced a case of a pregnant patient who had a congenital ATIII deficiency, which developed into severe VTE for the first time in her fourth pregnancy. We should take some heredity diseases into account in the case of severe VTE, even if they are multipara and have no family or medical history. In addition, when those who have hereditary thrombophilia disease with medication of NOAC get pregnant, they may not have to choose an elective pregnancy termination and be able to continue pregnancy.

## Figures and Tables

**Table 1a:** (a) Results of blood examination taken at the onset of VTE.

ATIII (%)	54.1

Protein S Activity (%)	33

Protein C Activity (%)	94

Anti-dsANA (titer)	<40

Anti-Cardiolipin (U/ml)	<1.2

MPO-ANCA (titer)	<40

**Table 1b:** (b) Results of blood examination taken after pregnancy.

	1 day after delivery	91 days after delivery
ATIII (%)	33.9	51.4
Protein S Activity (%)		75
